# Supplementary material for: From sequence to enzyme mechanism using multi-label machine learning
Source: BMC Bioinformatics. 2014 May 19;15:150. doi: 10.1186/1471-2105-15-150 (PMC4229970; doi:10.1186/1471-2105-15-150)
Supplement: Additional file 2 — Java code of ml2db. Additional file ml2db_code.tar.gz contains the Java source code to run the multi-label machine learning experiments and save the results to database. The code’s Javadoc is included. [file 1471-2105-15-150-S2.zip › additional file 2/ml2db/ml2db/doc/test/ml2db/dataharness/DataTableThreeTest.html]

DataTableThreeTest


JavaScript is disabled on your browser.


- Overview
- Package
- Class
- Use
- Tree
- Deprecated
- Index
- Help

- Prev Class
- Next Class

- Frames
- No Frames

- All Classes

- Summary:
- Nested |
- Field |
- Constr |
- Method

- Detail:
- Field |
- Constr |
- Method


test.ml2db.dataharness

## Class DataTableThreeTest

- java.lang.Object
- - junit.framework.Assert
  - - junit.framework.TestCase
    - - test.ml2db.dataharness.DataTableTest
      - - test.ml2db.dataharness.DataTableThreeTest

- All Implemented Interfaces:
  :   junit.framework.Test

  ---

    

  ```
  public class DataTableThreeTest
  extends DataTableTest
  ```

- - ### Field Summary

    Fields

    | Modifier and Type | Field and Description |
    | --- | --- |
    | `static java.lang.String` | `TABLE_THREE_NAME` |

    - ### Fields inherited from class test.ml2db.dataharness.DataTableTest

      `ATTRIBUTE_FIELD, CLASS_FIELD, INSTANCE_FIELD`
  - ### Constructor Summary

    Constructors

    Constructor and Description

    `DataTableThreeTest()`
  - ### Method Summary

    Methods

    | Modifier and Type | Method and Description |
    | --- | --- |
    | `java.lang.String` | `getInsertDataSqlStatement()` |
    | `void` | `testTableContent()` |

    - ### Methods inherited from class test.ml2db.dataharness.DataTableTest

      `createTestTable, getTable, getTableName, getTableReader, initialise, setTableName, testInstanceAttributeValueIsInTable, testTableInstanceAttribute, testTableInstanceClass`
    - ### Methods inherited from class junit.framework.TestCase

      `countTestCases, getName, run, run, runBare, setName, toString`
    - ### Methods inherited from class junit.framework.Assert

      `assertEquals, assertEquals, assertEquals, assertEquals, assertEquals, assertEquals, assertEquals, assertEquals, assertEquals, assertEquals, assertEquals, assertEquals, assertEquals, assertEquals, assertEquals, assertEquals, assertEquals, assertEquals, assertEquals, assertEquals, assertFalse, assertFalse, assertNotNull, assertNotNull, assertNotSame, assertNotSame, assertNull, assertNull, assertSame, assertSame, assertTrue, assertTrue, fail, fail, failNotEquals, failNotSame, failSame, format`
    - ### Methods inherited from class java.lang.Object

      `equals, getClass, hashCode, notify, notifyAll, wait, wait, wait`

- - ### Field Detail


    - #### TABLE\_THREE\_NAME

      ```
      public static java.lang.String TABLE_THREE_NAME
      ```
  - ### Constructor Detail


    - #### DataTableThreeTest

      ```
      public DataTableThreeTest()
      ```
  - ### Method Detail


    - #### getInsertDataSqlStatement

      ```
      public java.lang.String getInsertDataSqlStatement()
      ```

      **Overrides:**
      :   `getInsertDataSqlStatement` in class `DataTableTest`


    - #### testTableContent

      ```
      public void testTableContent()
      ```


- Overview
- Package
- Class
- Use
- Tree
- Deprecated
- Index
- Help

- Prev Class
- Next Class

- Frames
- No Frames

- All Classes

- Summary:
- Nested |
- Field |
- Constr |
- Method

- Detail:
- Field |
- Constr |
- Method
